# Comparing Different Doses of Intravenous Dexamethasone for Prolonging Analgesia After a Single-Shot Ultrasound-Guided Supraclavicular Brachial Plexus Block: A Prospective Randomized Study

**DOI:** 10.7759/cureus.80564

**Published:** 2025-03-14

**Authors:** Prasobh PV Mukundan, Kavya Rajendran, Tripti Saxena, Vinayakumar VS

**Affiliations:** 1 Critical Care, Epsom and St Helier University Hospitals, London, GBR; 2 General Medicine, Epsom and St Helier University Hospitals, London, GBR; 3 Anesthesiology, Apollo Hospitals, Bannerghatta Road, Bangalore, IND

**Keywords:** intravenous dexamethasone, peripheral nerve block analgesia, postoperative analgesia, ultrasound-guided regional anesthesia, ultrasound-guided supraclavicular brachial plexus block

## Abstract

Background

Supraclavicular brachial plexus block (SCBPB) is a widely used regional anesthesia technique for upper limb surgeries. Intravenous dexamethasone is known to prolong postoperative analgesia, but the optimal dose remains uncertain due to potential side effects like hyperglycemia associated with high doses. This study compares the efficacy of different doses of intravenous dexamethasone in enhancing the duration of analgesia following a single-shot ultrasound-guided SCBPB.

Methods

This prospective, randomized study included 60 American Society of Anesthesiologists (ASA) grade I-III patients undergoing upper limb surgery. All patients received an ultrasound-guided SCBPB, followed by general anesthesia (GA) and were randomly allocated into four groups using computer-generated randomization: group 1: 0.5% levobupivacaine (20 ml) + 8 mg IV dexamethasone; group 2: 0.5% levobupivacaine (20 ml) + 4 mg IV dexamethasone; group 3: 0.5% levobupivacaine (20 ml) + 2 mg IV dexamethasone; group 4 (control): 0.5% levobupivacaine (20 ml) + IV 0.9% normal saline.

Postoperative pain scores (visual analog scale, VAS), duration of analgesia, rescue analgesic requirement, incidence of nausea/vomiting, and blood glucose levels were recorded over 24 hours. Statistical analysis was performed using analysis of variance (ANOVA) and chi-square tests.

Results

Patients who received IV dexamethasone showed significantly prolonged analgesia compared to the control group (p < 0.001). The duration of analgesia in the 8 mg group was 13.8 ± 1.5 hours, compared to 11.2 ± 1.3 hours (4 mg), 9.4 ± 1.2 hours (2 mg), and 6.8 ± 1.1 hours in the control group. This represents a 103% increase in analgesia duration with 8 mg dexamethasone compared to the control.

The incidence of postoperative nausea and vomiting (PONV) was lower in dexamethasone groups (p = 0.02). However, 8 mg dexamethasone was associated with transient hyperglycemia (p = 0.04), with blood glucose levels peaking at 185 ± 12 mg/dL compared to 145 ± 10 mg/dL in the control group.

Conclusion

Intravenous dexamethasone significantly prolongs analgesia following SCBPB, with 8 mg providing the longest duration (103% increase vs. control). However, 4 mg may offer a balance between efficacy and minimal side effects, particularly in avoiding postoperative hyperglycemia.

## Introduction

Supraclavicular brachial plexus block (SCBPB) is a widely used regional anesthesia technique for upper limb surgeries, particularly for procedures on the elbow, forearm, wrist, and hand. It involves injecting a local anesthetic near the brachial plexus at the level of the first rib, blocking sensory and motor transmission to the upper limb. This technique provides effective intraoperative anesthesia and postoperative analgesia, reducing opioid consumption and associated side effects [1,2].

With advancements in regional anesthesia, ultrasound guidance has become the gold standard technique for performing SCBPB due to its superior accuracy and safety profile. Ultrasound enables real-time visualization of the brachial plexus, needle placement, and local anesthetic spread, thereby improving block success rates while minimizing complications such as vascular puncture, pneumothorax, and inadvertent nerve injury [3,4]. Compared to traditional landmark-based techniques, ultrasound-guided SCBPB provides higher success rates, requires lower volumes of local anesthetic, and has been associated with reduced complication rates, particularly in preventing inadvertent pneumothorax and vascular injury [4]. The ability to visualize the needle tip in real time ensures optimal needle placement and precise perineural drug deposition, making it the preferred approach in modern regional anesthesia practice.

Despite its effectiveness, a single-shot SCBPB is limited by the duration of action of the local anesthetic. Various adjuvants have been explored to prolong analgesia, including opioids, alpha-2 agonists, magnesium, and corticosteroids [5,6]. Among these, dexamethasone, a synthetic corticosteroid with potent anti-inflammatory and analgesic properties, has shown promising results [7,8].

Dexamethasone can be administered perineurally (directly with the local anesthetic injection) or intravenously. While perineural administration may provide longer analgesia, concerns remain regarding potential neurotoxicity, impaired wound healing, increased infection risk, and its off-label use [9,10]. Intravenous dexamethasone offers a safer and equally effective alternative by modulating systemic inflammation and reducing pain transmission. However, the optimal intravenous dose required to maximize analgesia duration while minimizing systemic side effects such as hyperglycemia and other corticosteroid-related adverse effects remains unclear [11,12].

This study aims to compare the efficacy of varying doses of intravenous dexamethasone in prolonging analgesia following a single-shot ultrasound-guided SCBPB. We hypothesized that higher doses would be associated with prolonged analgesia but might also lead to increased side effects.

This research was previously presented as an e-research paper at the 68th Annual Conference of the Indian Society of Anaesthesiologists (ISACON 2021), held in Ahmedabad from November 24 to 28, 2021, highlighting its relevance to the anesthesia and regional block community [13].

## Materials and methods

Study design and setting

This randomized, prospective, controlled study was conducted in the Department of Anesthesiology and Critical Care at Apollo Hospitals, Bangalore. The study received approval from the Institutional Ethics Committee on 26th October 2018, and written informed consent was obtained from all participants. Data collection commenced in November 2018 and continued until April 2020.

Study population and sample size

A total of 60 American Society of Anesthesiologists (ASA) grade I-III patients scheduled for various elective upper limb surgeries, primarily open reduction and internal fixation (ORIF) of fractures, under ultrasound-guided SCBPB were included in the study. While surgical duration may influence the total time of block effect, all patients received a standardized ultrasound-guided SCBPB with 20 mL of 0.5% levobupivacaine, ensuring uniformity in block characteristics across groups. Participants were randomly assigned to one of the four groups, with 15 patients in each group, using a computerized randomization method, ensuring allocation concealment and minimizing selection bias. Group 1 received 0.5% levobupivacaine (20 mL) with 8 mg IV dexamethasone, group 2 received 0.5% levobupivacaine (20 mL) with 4 mg IV dexamethasone, group 3 received 0.5% levobupivacaine (20 mL) with 2 mg IV dexamethasone, while group 4 (control) received 0.5% levobupivacaine (20 mL) with 0.9% IV normal saline (20 mL).

Inclusion and exclusion criteria

The inclusion criteria for this study consisted of ASA grade I-III patients aged 18 years or older who were scheduled for upper limb surgeries below the mid-humerus. Patients with ASA grade IV or higher, coagulopathy, known allergies to local anesthetics, infection at the injection site, or diabetes mellitus (to avoid confounding effects on glucose levels) were excluded from the study (Table 1).

**Table 1:** Inclusion and exclusion criteria for study participants. ASA: American Society of Anesthesiologists classification.

Inclusion criteria	Exclusion criteria
ASA I-III	ASA IV and above
Age ≥18 years	Coagulopathy
Scheduled for upper limb surgeries below the mid-humerus	Allergy to local anesthetics
	Infection at the injection site
	Diabetes mellitus (to avoid confounding effects on glucose levels)

Methodology

All patients underwent a preoperative assessment, including a detailed medical history, physical examination, and standard laboratory investigations.

Preoperative Management

Standard monitoring was conducted using ECG, heart rate, non-invasive blood pressure (NIBP), pulse oximetry (oxygen saturation), and end-tidal CO₂. Patients received IV pantoprazole (40 mg) and IV ondansetron (4 mg) as premedication. Preoperative blood glucose levels were measured using venous blood samples, which were sent to the laboratory for analysis to ensure accuracy and standardization before shifting to the operating room.

Regional Block and General Anesthesia

All patients underwent an ultrasound-guided SCBPB using a single-point injection technique with a 21-gauge, short-bevel, insulated needle. A total volume of 20 mL of 0.5% levobupivacaine was administered. Each group received its designated IV dexamethasone dose according to the randomization.

Following the block, patients received general anesthesia with an induction regimen consisting of IV midazolam (0.05 mg/kg), IV fentanyl (2 mcg/kg), IV propofol (2 mg/kg), and IV atracurium (0.5 mg/kg) for muscle relaxation. Anesthesia was maintained using oxygen/nitrous oxide/sevoflurane at a minimum alveolar concentration (MAC) of 0.9-1.1. General anesthesia was used for standardization to ensure uniform intraoperative conditions across all patients. Induction and maintenance were performed with fixed weight-based doses, and fentanyl (2 mcg/kg) was administered uniformly for induction. The regional block served as the primary source of intraoperative analgesia, with no additional opioids administered during surgery.

At the end of surgery, neuromuscular blockade was reversed with IV neostigmine (0.05 mg/kg) and IV glycopyrrolate (0.01 mg/kg), followed by extubation and transfer to the post-anesthesia care unit (PACU).

Outcome measures

The primary outcome of this study was the total duration of postoperative analgesia, defined as the time from block administration to the first request for rescue analgesia. Secondary outcomes included postoperative blood glucose levels, which were measured at four time points: preoperative, intraoperative (one hour after IV dexamethasone or saline administration to ensure consistency across all participants), immediate postoperative, and six hours postoperative. The incidence of postoperative nausea and vomiting (PONV) was also recorded. Pain assessment was conducted using the visual analog scale (VAS; 0-10) at the following time points: immediate postoperative period and at one, two, six, 12, and 24 hours. Rescue analgesia was administered according to the following protocol: IV paracetamol (1 g) was the first-line agent. If the VAS score remained ≥4 after paracetamol, IV tramadol (50 mg) was given. The study concluded once the first dose of rescue analgesia was administered.

Sample size calculation

The required sample size was determined based on mean VAS scores from a previous study (Dhanger et al.), which reported analgesia durations of 11.88 ± 1.31 hours versus 6.47 ± 0.93 hours in different groups [14]. Using these values, a formal power analysis was conducted, selecting a 99% confidence level and 90% power, leading to a final sample size of 60 patients (15 per group), which was deemed sufficient to detect a statistically significant difference.

Statistical analysis

Data were analyzed using SPSS version 22 (IBM Corp., Armonk, NY). Categorical data were presented as frequencies and proportions, while continuous data were expressed as mean and standard deviation. The chi-square test was used for categorical variables, while analysis of variance (ANOVA) was used to compare continuous variables across the four groups. A p-value < 0.05 was considered statistically significant.

## Results

Demographic data

The mean age of the participants in group 1 was 37.67 ± 12.65 years, in group 2 was 36.33 ± 14.03 years, in group 3 was 43.47 ± 11.06 years, and in group 4 was 41.40 ± 12.75 years. There was no statistically significant difference in age distribution among the four groups. Similarly, the sex distribution among the four groups showed no significant difference. In groups 1 and 2, 26.67% were females, and 73.33% were males. In groups 3 and 4, 46.67% were females, and 53.33% were males. The ASA classification was comparable across groups, with no significant difference observed. The majority of patients in all four groups were ASA I, accounting for 80.00% in groups 1 and 2 and 86.67% in groups 3 and 4.

Hemodynamic parameters

Heart rate measurements at various time intervals showed no statistically significant differences between the four groups. Similarly, systolic blood pressure (SBP) values showed significant differences at the 60-minute and 90-minute marks (p = 0.045 and p = 0.014, respectively), while no significant differences were observed at other intervals (Table 2).

**Table 2:** Mean SBP comparison between the four groups at different intervals of time. Data are presented as mean ± standard deviation (SD). A one-way ANOVA test was used to analyze differences in systolic blood pressure (SBP) between the four groups at different time intervals. The F-values corresponding to the ANOVA test are provided in the second-last column, and the p-values are listed in the last column. Statistically significant differences (p < 0.05) are marked with an asterisk (*). For time intervals where only a single data point was available (n = 1), SD could not be calculated and is marked as "Not applicable (n = 1)". SBP: systolic blood pressure; SD: standard deviation; F-value: test statistic from one-way ANOVA; P-value: probability value.

Time interval	Group 1 (Mean ± SD)	Group 2 (Mean ± SD)	Group 3 (Mean ± SD)	Group 4 (Mean ± SD)	Total (Mean ± SD)	F-value (ANOVA)	P-value
Baseline	118.07 ± 7.97	120.27 ± 8.17	121.20 ± 7.12	118.80 ± 8.58	119.58 ± 7.87	0.35	0.704
5 minutes	120.47 ± 9.33	120.13 ± 10.21	118.20 ± 8.14	117.80 ± 8.57	119.15 ± 8.94	0.29	0.805
10 minutes	117.33 ± 8.20	117.07 ± 9.74	117.20 ± 9.85	117.47 ± 9.36	117.27 ± 9.07	0.12	0.999
15 minutes	117.67 ± 7.22	116.80 ± 8.97	114.00 ± 6.68	116.87 ± 8.64	116.33 ± 7.86	0.22	0.609
30 minutes	113.87 ± 8.57	116.73 ± 8.37	116.20 ± 8.54	114.73 ± 8.46	115.38 ± 8.34	0.41	0.781
45 minutes	114.53 ± 9.15	115.87 ± 7.23	118.40 ± 8.69	119.20 ± 8.51	117.00 ± 8.43	0.58	0.401
60 minutes	112.07 ± 8.85	116.20 ± 8.82	118.80 ± 7.23	120.67 ± 9.12	116.93 ± 8.93	3.21*	0.045*
75 minutes	114.07 ± 8.27	115.73 ± 7.96	118.13 ± 6.07	117.73 ± 6.92	116.42 ± 7.35	0.65	0.406
90 minutes	113.47 ± 6.86	120.77 ± 6.51	121.08 ± 8.55	120.31 ± 5.88	118.70 ± 7.57	4.03*	0.014*
105 minutes	118.17 ± 6.18	122.88 ± 11.08	122.67 ± 7.00	121.33 ± 6.15	120.78 ± 7.73	0.78	0.524
120 minutes	117.67 ± 7.94	121.00 ± 8.41	122.00 ± 7.83	122.50 ± 5.00	120.44 ± 7.11	0.39	0.728
135 minutes	118.33 ± 8.62	120.00 ± Not applicable (n = 1)	130.00 ± Not applicable (n = 1)	130.00 ± Not applicable (n = 1)	122.50 ± 7.99	0.42	0.609
150 minutes	123.00 ± 4.24	-	-	126.00 ± Not applicable (n = 1)	124.00 ± 3.46	0.53	0.667
165 minutes	-	-	-	120.00 ± Not applicable (n = 1)	120.00 ± Not applicable (n = 1)	-	-
180 minutes	-	-	-	-	-	-	-
Postoperative	124.80 ± 8.28	122.53 ± 8.47	126.53 ± 5.83	124.13 ± 4.93	124.50 ± 7.01	0.68	0.484

**Figure 1:**
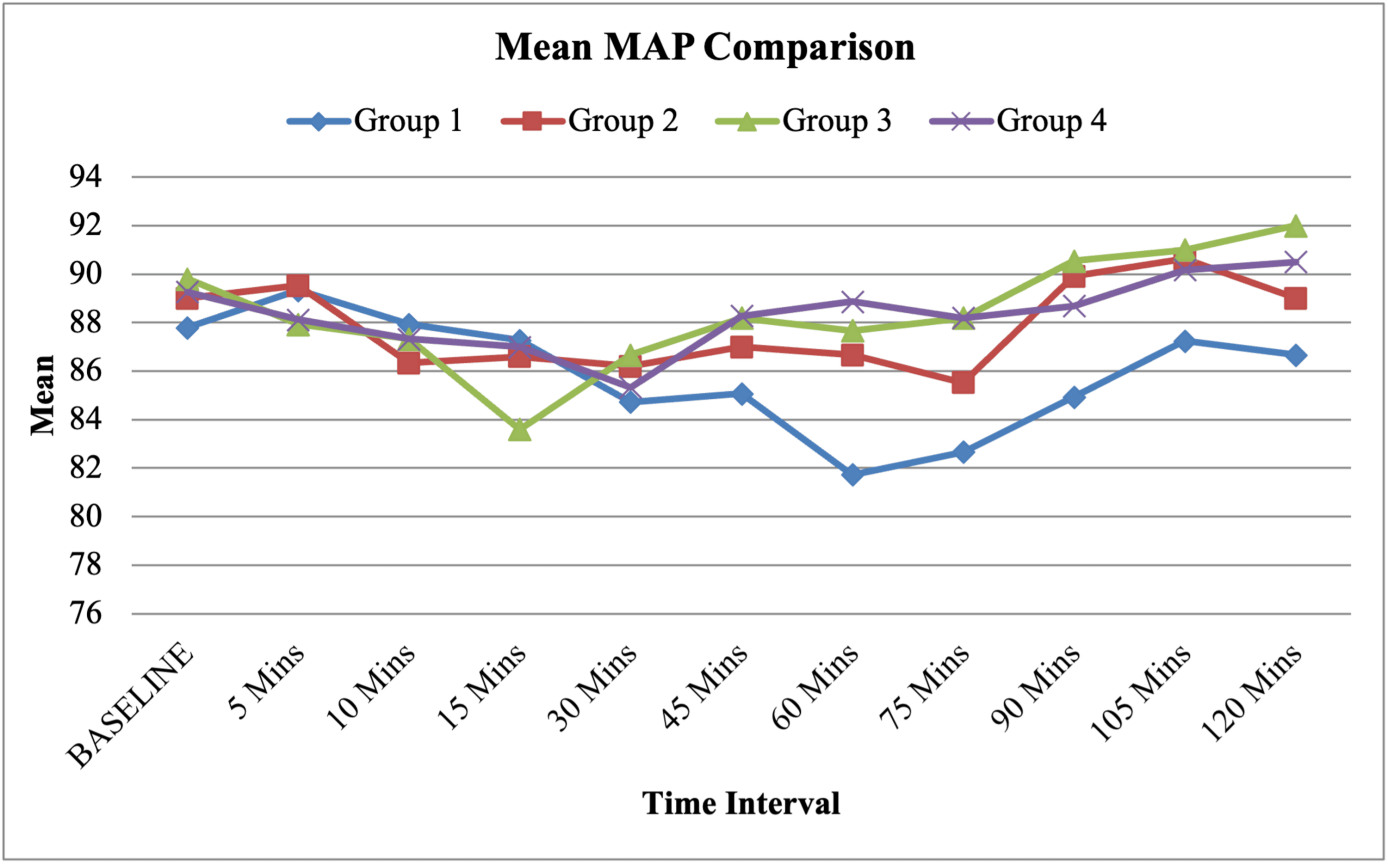
Line diagram showing the mean MAP comparison between the four groups at different intervals of time. Mean arterial pressure (MAP) was comparable at most time points but showed statistically significant differences between 60 and 90 minutes (p = 0.03 to p = 0.036).

Diastolic blood pressure (DBP) comparison showed a significant difference at the 75-minute interval (p = 0.004) but was otherwise comparable between the groups (Table 3). Mean arterial pressure (MAP) showed significant differences between 60 and 90 minutes (p = 0.03 and p = 0.036) but was comparable at other time points (Figure 1).

**Table 3:** Mean DBP comparison between the four groups at different intervals of time. Data are presented as mean ± standard deviation (SD). A one-way ANOVA test was used to analyze differences in diastolic blood pressure (DBP) between the four groups at different time intervals. The F-values corresponding to the ANOVA test are provided in the second-last column, and the p-values are listed in the last column. Statistically significant differences (p < 0.05) are marked with an asterisk (*). For time intervals where only a single data point was available (n = 1), SD could not be calculated and is marked as "Not applicable (n = 1)". DBP: diastolic blood pressure; SD: standard deviation; F-value: test statistic from one-way ANOVA; P-value: probability value.

Time Interval	Group 1 (Mean ± SD)	Group 2 (Mean ± SD)	Group 3 (Mean ± SD)	Group 4 (Mean ± SD)	Total (Mean ± SD)	F-value (ANOVA)	P-value
Baseline	72.80 ± 4.33	72.93 ± 5.75	74.00 ± 5.76	74.53 ± 7.54	73.57 ± 5.85	0.21	0.827
5 minutes	73.73 ± 5.30	74.27 ± 8.21	72.93 ± 8.03	73.47 ± 8.16	73.60 ± 7.35	0.32	0.970
10 minutes	73.47 ± 5.32	71.07 ± 7.36	72.40 ± 8.39	72.27 ± 7.44	72.30 ± 7.08	0.15	0.841
15 minutes	72.20 ± 3.47	71.73 ± 6.54	68.67 ± 6.83	71.73 ± 7.09	71.08 ± 6.17	0.27	0.379
30 minutes	70.20 ± 4.87	70.93 ± 8.17	71.07 ± 7.00	70.80 ± 6.18	70.75 ± 6.50	0.18	0.985
45 minutes	70.27 ± 5.56	72.67 ± 6.40	72.67 ± 7.99	72.53 ± 6.86	72.03 ± 6.67	0.42	0.714
60 minutes	66.73 ± 5.08	70.93 ± 6.67	72.27 ± 7.78	72.80 ± 6.75	70.68 ± 6.90	2.91	0.063
75 minutes	66.93 ± 5.75	70.53 ± 4.93	73.27 ± 5.86	73.47 ± 4.37	71.05 ± 5.78	4.21*	0.004*
90 minutes	70.67 ± 4.94	74.62 ± 6.85	74.31 ± 5.71	73.08 ± 5.33	73.07 ± 5.78	0.98	0.253
105 minutes	71.75 ± 5.31	74.63 ± 9.02	75.33 ± 4.13	74.33 ± 4.27	73.63 ± 6.03	0.55	0.605
120 minutes	71.17 ± 5.71	73.50 ± 5.26	77.00 ± 3.46	74.50 ± 5.26	73.72 ± 5.14	0.49	0.388
135 minutes	71.67 ± 7.64	80.00 ± Not applicable (n = 1)	78.00 ± Not applicable (n = 1)	80.00 ± Not applicable (n = 1)	75.50 ± 6.44	0.58	0.710
150 minutes	71.00 ± 1.41	-	-	78.00 ± Not applicable (n = 1)	73.33 ± 4.16	0.61	0.154
165 minutes	-	-	-	78.00 ± Not applicable (n = 1)	78.00 ± Not applicable (n = 1)	-	-
180 minutes	-	-	-	-	-	-	-
Postoperative	73.67 ± 6.09	76.33 ± 7.96	78.93 ± 4.71	77.20 ± 6.04	76.53 ± 6.44	0.74	0.154

Pain assessment and analgesic requirements

Postoperative pain scores, measured using the VAS, showed a significant difference at six and 12 hours among the four groups (p < 0.001). At other time intervals, no significant differences were observed (Table 4). The mean time to first rescue analgesia was significantly prolonged in group 1 (835 ± 66.55 minutes) compared to group 4 (427 ± 57.31 minutes), with a p-value of <0.001 (Table 5).

**Table 4:** VAS distribution between the four groups at different intervals of time. Data are presented as absolute counts (N) and percentages (%). The chi-square (χ²) test was used to compare differences in the visual analog scale (VAS) distribution between the four groups at different time intervals. The chi-square values (χ²) and degrees of freedom (df) are provided in the second-last column, and the p-values are listed in the last column. Statistically significant differences (p < 0.05) are marked with an asterisk (*). VAS: visual analog scale; N: number of patients; χ²: chi-square test statistic; df: degrees of freedom; P-value: probability value.

Time interval	VAS score	Group 1 (N, %)	Group 2 (N, %)	Group 3 (N, %)	Group 4 (N, %)	Chi-square (x²)	df (Degrees of freedom)	P-value
Immediate postoperative	3	15 (100.0%)	15 (100.0%)	15 (100.0%)	15 (100.0%)	-	-	-
1 hour	3	15 (100.0%)	15 (100.0%)	15 (100.0%)	15 (100.0%)	-	-	-
2 hours	3	15 (100.0%)	15 (100.0%)	15 (100.0%)	15 (100.0%)	-	-	-
6 hours	3	15 (100.0%)	15 (100.0%)	13 (86.7%)	3 (20.0%)	36.894	3	<0.001*
6 hours	4	0 (0.0%)	0 (0.0%)	2 (13.3%)	12 (80.0%)	-	-	-
12 hours	3	15 (100.0%)	0 (0.0%)	0 (0.0%)	0 (0.0%)	65.122	6	<0.001*
12 hours	3	0 (0.0%)	15 (100.0%)	14 (93.3%)	12 (80.0%)	-	-	-
12 hours	3	0 (0.0%)	0 (0.0%)	1 (6.7%)	3 (20.0%)	-	-	-
24 hours	4	11 (73.3%)	11 (73.3%)	12 (80.0%)	13 (86.7%)	1.08	3	0.782
24 hours	5	4 (26.7%)	4 (26.7%)	3 (20.0%)	2 (13.3%)	-	-	-

**Table 5:** Mean time of rescue analgesic comparison between the four groups. Data are presented as mean ± standard deviation (SD). A one-way ANOVA test was used to compare the time to rescue analgesia between the four groups. The F-value corresponding to the ANOVA test is provided in a separate row at the bottom, along with the p-value. Statistically significant differences (p < 0.05) are marked with an asterisk (*). SD: standard deviation; F-value: test statistic from one-way ANOVA; P-value: probability value.

Group	Mean time of rescue analgesic in minutes (Mean ± SD)	F-value (ANOVA)	P-value
Group 1	835.00 ± 66.55	-	-
Group 2	658.33 ± 62.50	-	-
Group 3	547.33 ± 53.48	-	-
Group 4	427.00 ± 57.31	-	-
Total	616.92 ± 162.37	-	-
ANOVA test	-	18.72*	<0.001*

The distribution of rescue analgesic use showed a significant difference between six and 24 hours (p < 0.001). Group 4 required the highest percentage of rescue analgesics at the six-hour mark (Table 6).

**Table 6:** Rescue analgesics distribution between the four groups at different intervals of time. Data are presented as absolute counts (N) and percentages (%). The chi-square (χ²) test was used to compare differences in rescue analgesics distribution between the four groups at different time intervals. The chi-square values (χ²) and degrees of freedom (df) are provided in the second-last column, and the p-values are listed in the last column. Statistically significant differences (p < 0.05) are marked with an asterisk (*). "NIL" indicates that no rescue analgesic was administered during that specific time interval. N: number of patients; χ²: chi-square test statistic; df: degrees of freedom; P-value: probability value.

Time interval	Group 1 (N, %)	Group 2 (N, %)	Group 3 (N, %)	Group 4 (N, %)	Chi-square (χ²)	df (Degrees of freedom)	P-value
Immediate postoperative	15 (100.0%)	15 (100.0%)	15 (100.0%)	15 (100.0%)	-	-	-
1 hour	15 (100.0%)	15 (100.0%)	15 (100.0%)	15 (100.0%)	-	-	-
2 hours	15 (100.0%)	15 (100.0%)	15 (100.0%)	15 (100.0%)	-	-	-
6 hours (Paracetamol 1 gm IV)	0 (0.0%)	0 (0.0%)	2 (13.3%)	12 (80.0%)	36.894	3	<0.001*
6 hours (NIL)	15 (100.0%)	15 (100.0%)	13 (86.7%)	3 (20.0%)	-	-	-
12 hours (Paracetamol 1 gm IV & Tramadol 50 mg IV)	0 (0.0%)	0 (0.0%)	15 (100.0%)	15 (100.0%)	120.000	6	<0.001*
12 hours (Paracetamol 1 gm IV)	0 (0.0%)	15 (100.0%)	0 (0.0%)	0 (0.0%)	-	-	-
12 hours (NIL)	15 (100.0%)	0 (0.0%)	0 (0.0%)	0 (0.0%)	-	-	-
24 hours (Paracetamol 1 gm IV)	15 (100.0%)	0 (0.0%)	0 (0.0%)	0 (0.0%)	60.000	3	<0.001*
24 hours (Paracetamol 1 gm IV & Tramadol 50 mg IV)	0 (0.0%)	15 (100.0%)	15 (100.0%)	15 (100.0%)	-	-	-

Blood glucose levels

**Figure 2:**
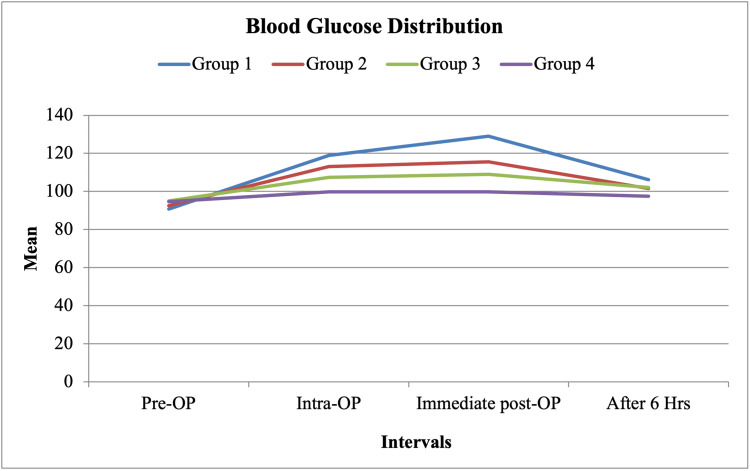
Blood glucose distribution across different time intervals. Blood glucose levels varied significantly across all measured time points (p < 0.001). Group 1 exhibited the highest intraoperative and postoperative glucose levels, while group 4 had the lowest levels at all time intervals. The most significant differences were observed in the intraoperative and immediate postoperative periods. Data are presented as mean values for each group.

Blood glucose levels varied significantly at all measured intervals (p < 0.001). Intraoperatively and at six hours postoperatively, glucose levels were significantly lower in group 4 compared to the other three groups. In contrast, group 1 exhibited the highest intraoperative and postoperative glucose levels (Table 7 and Figure 2).

**Table 7:** Blood glucose distribution between the four groups at different intervals of time. Data are presented as mean ± standard deviation (SD). A one-way ANOVA test was used to compare the mean blood glucose levels across the four groups at different time intervals. The F-value and corresponding p-value are presented in the last two columns. Statistically significant differences (p < 0.05) are marked with an asterisk (*). SD: standard deviation; F-value: test statistic from one-way ANOVA; P-value: probability value.

Time interval	Group 1 (Mean ± SD)	Group 2 (Mean ± SD)	Group 3 (Mean ± SD)	Group 4 (Mean ± SD)	Total (Mean ± SD)	F-value (ANOVA)	P-value
Preoperative	90.80 ± 4.06	92.53 ± 5.05	95.00 ± 3.91	94.73 ± 4.91	93.27 ± 4.72	2.95*	0.043*
Intraoperative	118.93 ± 6.86	113.00 ± 6.69	107.47 ± 2.90	99.87 ± 4.16	109.82 ± 8.84	12.87*	<0.001*
Immediate postoperative	128.93 ± 4.42	115.60 ± 4.61	109.00 ± 4.04	99.80 ± 5.86	113.33 ± 11.67	18.42*	<0.001*
After 6 hours	106.20 ± 8.19	101.53 ± 4.70	102.00 ± 3.95	97.47 ± 4.47	101.80 ± 6.27	5.21*	0.001*
ANOVA test	-	-	-	-	-	-	-

Postoperative nausea and vomiting

**Figure 3:**
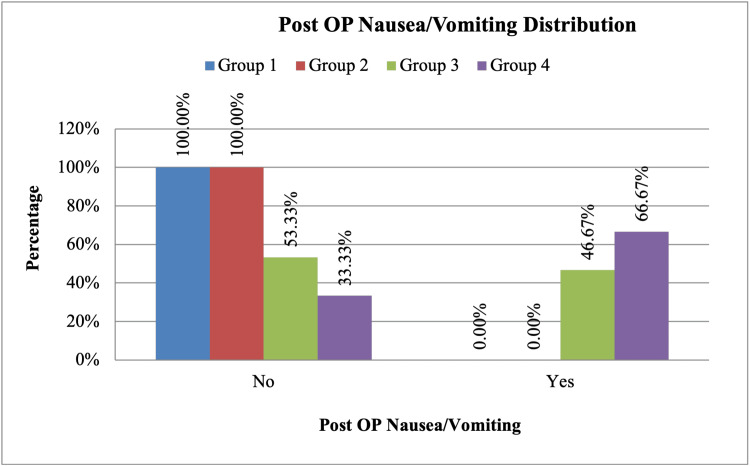
Bar diagram showing the postoperative nausea/vomiting (PONV) distribution between the four groups. PONV incidence differed significantly among groups (p < 0.001), with 46.67% in group 3 and 66.67% in group 4.

A significant difference in PONV distribution was observed, with group 3 experiencing PONV in 46.67% of cases and group 4 in 66.67% of cases (p < 0.001) (Figure 3). Further analysis of nausea severity showed that group 4 had the highest incidence of moderate (20%) and severe (6.67%) nausea (Table 8).

**Table 8:** Grading of nausea/vomiting distribution between the four groups. Data are presented as absolute counts (N) and percentages (%). A chi-square (χ²) test was used to compare the grading of nausea and vomiting between the four groups. The chi-square value (χ²), degrees of freedom (df), and corresponding p-values are reported in the final row. Statistically significant differences (p < 0.05) are marked with an asterisk (*). N: number of patients; χ²: chi-square test statistic; df: degrees of freedom; P-value: probability value.

Grading of nausea/vomiting	Group 1 (N, %)	Group 2 (N, %)	Group 3 (N, %)	Group 4 (N, %)	Total (N, %)
Mild	0 (0.0%)	0 (0.0%)	7 (46.7%)	6 (40.0%)	13 (21.7%)
Moderate	0 (0.0%)	0 (0.0%)	0 (0.0%)	3 (20.0%)	3 (5.0%)
Nil	15 (100.0%)	15 (100.0%)	8 (53.3%)	5 (33.3%)	43 (71.7%)
Severe	0 (0.0%)	0 (0.0%)	0 (0.0%)	1 (6.7%)	1 (1.7%)
Chi-square (χ²)	-	-	-	-	32.293
Degrees of freedom (df)	-	-	-	-	9
P-value	-	-	-	-	<0.001*

The number of nausea episodes did not show significant variation across groups (p = 0.384), with only one patient in group 4 experiencing three episodes (Table 9).

**Table 9:** Number of episodes distribution between the four groups. Data are presented as absolute counts (N) and percentages (%). A chi-square (χ²) test was used to compare the distribution of episodes between the four groups. The chi-square value (χ²), degrees of freedom (df), and corresponding p-values are reported in the final row. Statistically significant differences (p < 0.05) are marked with an asterisk (*). N: number of patients; χ²: chi-square test statistic; df: degrees of freedom; P-value: probability value.

Number of Episodes	Group 1 (N, %)	Group 2 (N, %)	Group 3 (N, %)	Group 4 (N, %)	Total (N, %)
3 Episodes	0 (0.0%)	0 (0.0%)	0 (0.0%)	1 (6.67%)	1 (1.67%)
Nil	15 (100.0%)	15 (100.0%)	15 (100.0%)	14 (93.33%)	59 (98.33%)
Chi-square (χ²)	-	-	-	-	3.051
Degrees of Freedom (df)	-	-	-	-	3
P-Value	-	-	-	-	0.384

Treatment for PONV

The administration of ondansetron (Emeset 4 mg IV) was significantly higher in groups 3 and 4 (46.67% and 66.67%, respectively), indicating a higher requirement for antiemetics in these groups (p < 0.001) (Table 10).

**Table 10:** Treatment distribution between the four groups. Data are presented as absolute counts (N) and percentages (%). A chi-square (χ²) test was used to compare the distribution of treatment between the four groups. The chi-square value (χ²), degrees of freedom (df), and corresponding p-values are reported in the final row. Statistically significant differences (p < 0.05) are marked with an asterisk (*). N: number of patients; χ²: chi-square test statistic; df: degrees of freedom; P-value: probability value.

Treatment	Group 1 (N, %)	Group 2 (N, %)	Group 3 (N, %)	Group 4 (N, %)	Total (N, %)
Inj. ondansetron 4 mg IV	0 (0.0%)	0 (0.0%)	7 (46.67%)	10 (66.67%)	17 (28.33%)
NIL	15 (100.0%)	15 (100.0%)	8 (53.33%)	5 (33.33%)	43 (71.67%)
Chi-square (χ²)	-	-	-	-	25.198
Degrees of freedom (df)	-	-	-	-	3
P-value	-	-	-	-	<0.001*

Summary

Dexamethasone demonstrated a dose-dependent effect in prolonging postoperative analgesia, with higher doses significantly delaying the need for rescue analgesia. However, increased doses were associated with a transient rise in blood glucose levels, with group 1 showing the highest values intraoperatively and postoperatively. Hemodynamic parameters remained stable across all groups, with no clinically significant variations in heart rate, SBP, or MAP. Postoperative nausea and vomiting were more frequent in groups 3 and 4, requiring increased antiemetic intervention. These findings suggest that while dexamethasone effectively enhances postoperative analgesia, the dose-dependent metabolic and gastrointestinal effects should be considered in clinical decision-making.

## Discussion

This study confirmed that intravenous dexamethasone significantly prolongs the duration of analgesia in a dose-dependent manner following ultrasound-guided SCBPB. The 8 mg dexamethasone group exhibited the longest analgesia duration (835 ± 66.55 minutes, p < 0.001), followed by the 4 mg (658.33 ± 62.50 minutes) and 2 mg (547.33 ± 53.48 minutes) groups, with the control group having the shortest duration (427 ± 57.31 minutes). Patients receiving dexamethasone required significantly fewer rescue analgesics, particularly in the 8 mg group, where requirements were minimal within the first 24 hours postoperatively. Hemodynamic parameters remained stable across all groups, with no clinically significant variations. While 8 mg dexamethasone led to a significant increase in blood glucose levels (peaking at 128.93 ± 4.42 mg/dL, p < 0.001), no patients required insulin therapy or prolonged hospital stays due to hyperglycemia. Additionally, dexamethasone demonstrated strong antiemetic effects, with the 8 mg and 4 mg groups having no reported cases of PONV, compared to a 46.7% incidence in the 2 mg group and 66.7% in the control group (p < 0.001). These findings suggest that 4 mg IV dexamethasone may provide an optimal balance between prolonged analgesia, reduced opioid consumption, and minimal metabolic side effects.

SCBPB is an established regional anesthesia technique for upper limb surgeries, providing effective intraoperative anesthesia and postoperative analgesia while reducing opioid consumption and its associated side effects [2,15]. The compact arrangement of the brachial plexus at the level of the trunks (C5-T1) allows for dense and reliable anesthesia with a single injection, making it a preferred technique [16]. The introduction of ultrasound guidance has significantly enhanced the accuracy of this block and improved success rates while minimizing complications such as vascular puncture and pneumothorax [3,4].

Despite its effectiveness, a single-shot SCBPB is inherently limited by the pharmacokinetics of the local anesthetic used. Various adjuvants, including opioids, alpha-2 agonists, magnesium, and corticosteroids, have been studied for their potential to prolong the duration of analgesia [5,6]. Among these, dexamethasone has shown significant promise due to its potent anti-inflammatory and analgesic properties [8].

Dexamethasone can be administered perineurally or intravenously. Perineural administration has been reported to provide extended analgesia; however, concerns regarding potential neurotoxicity, impaired wound healing, and an increased risk of infection have limited its widespread adoption [9]. Recent evidence suggests that intravenous dexamethasone is equally effective in prolonging analgesia without the associated neurotoxic risks [10,17]. However, the optimal intravenous dose for maximizing analgesic duration while minimizing systemic side effects remains uncertain.

In this study, three different doses of intravenous dexamethasone (8 mg, 4 mg, and 2 mg) were compared with a control group in patients undergoing upper limb surgery under ultrasound-guided SCBPB. The results confirmed that intravenous dexamethasone significantly prolongs the duration of analgesia in a dose-dependent manner. The 8 mg dexamethasone group exhibited the longest duration of analgesia (835 ± 66.55 minutes, p < 0.001), followed by the 4 mg (658.33 ± 62.50 minutes) and 2 mg (547.33 ± 53.48 minutes) groups. The control group had the shortest analgesia duration (427 ± 57.31 minutes). These findings align with a previous study by Desmet et al., who reported similar results regarding prolonged analgesia with intravenous dexamethasone [7].

The need for rescue analgesia was significantly lower in groups receiving IV dexamethasone, particularly in the 8 mg group, where requirements were minimal in the first 24 hours postoperatively. These results are consistent with previous studies, such as those by Dhanger et al., which demonstrated that even low-dose IV dexamethasone (2 mg) can effectively prolong analgesia and reduce opioid consumption [14].

Hemodynamic parameters remained stable across all groups, with minor, non-clinically significant variations in SBP, DBP, and MAP at 60-90 minutes. These variations were likely due to hemodynamic responses during extubation rather than a direct effect of dexamethasone [12].

One of the main concerns with corticosteroid use is the risk of hyperglycemia. In this study, 8 mg IV dexamethasone significantly increased blood glucose levels intraoperatively and postoperatively, peaking at 128.93 ± 4.42 mg/dL (p < 0.001). This is consistent with previous studies by Hans et al. and Ho et al., who demonstrated a dose-dependent increase in blood glucose levels following IV dexamethasone administration [10,11]. However, none of the patients in this study required insulin therapy or experienced prolonged hospital stays due to hyperglycemia. The 4 mg dose provided an optimal balance between analgesic benefits and minimal metabolic effects, making it a preferable choice in patients at risk of hyperglycemia.

In addition to its analgesic effects, dexamethasone is well known for its antiemetic properties. The results of this study showed that IV dexamethasone significantly reduced the incidence of PONV. The 8 mg and 4 mg groups had no reported cases of PONV, whereas the 2 mg group had a 46.7% incidence, and the control group had a 66.7% incidence (p < 0.001). These findings agree with studies by Gan et al., which highlighted the antiemetic benefits of dexamethasone, particularly in reducing opioid-induced nausea [12].

Clinical implications and future directions

Our findings indicate that IV dexamethasone is a valuable adjuvant for prolonging analgesia following SCBPB. While 8 mg provides the longest duration, it is associated with transient hyperglycemia, suggesting that 4 mg may be the optimal dose for balancing efficacy and safety. Further studies on the long-term metabolic effects of repeated IV dexamethasone doses in surgical patients are warranted.

Limitations of the study

Although this study demonstrates the efficacy of intravenous dexamethasone in prolonging analgesia following SCBPB, certain limitations must be acknowledged. The relatively small sample size (n = 60) may limit the generalizability of the findings to a larger population. Additionally, BMI was not specifically analyzed, making it uncertain whether the findings are applicable to obese patients. The study was limited to ASA I-III patients, excluding ASA IV and diabetic patients to minimize confounding effects on glucose levels. Future studies may consider including diabetic patients with controlled glucose levels to assess safety in this subgroup. Furthermore, the follow-up period was limited to 24 hours postoperatively, meaning long-term effects of dexamethasone on pain relief, hyperglycemia, and other corticosteroid-related metabolic effects were not assessed. Future research should include larger sample sizes, a broader patient population, and an extended follow-up period to further establish the safety and efficacy of intravenous dexamethasone in this context.

## Conclusions

This study demonstrates that intravenous dexamethasone prolongs postoperative analgesia following SCBPB in a dose-dependent manner. While higher doses provided extended pain relief, they were associated with transient hyperglycemia, highlighting a trade-off between efficacy and metabolic side effects. Among the doses studied, 4 mg appeared to strike the optimal balance, offering significant analgesic benefits while minimizing adverse effects, including a reduced incidence of PONV. Clinically, 4 mg of intravenous dexamethasone may serve as the preferred dose for routine use, whereas 8 mg should be reserved for cases requiring prolonged analgesia despite the risk of hyperglycemia. Future studies with larger cohorts and extended follow-up periods are needed to further validate these findings and refine the optimal use of intravenous dexamethasone in regional anesthesia.

## Appendices

Study proforma

Patient Information

**Table 11:** Patient information. ASA: American Society of Anesthesiologists.

Patient ID	
Group (I/II/III/IV)	
ASA grade	
Time of block administration	
Time of dexamethasone administration	
Time of induction of general anesthesia	

Intraoperative Monitoring

**Table 12:** Intraoperative monitoring. HR: heart rate; BP: blood pressure; MAP: mean arterial pressure.

Time (minutes)	Baseline		5		10		15	30	45		60		75		90
HR															
BP															
MAP															
Time (minutes)		105		120		135		150		165		180		Postoperative	
HR															
BP															
MAP															

Postoperative Plan

**Table 13:** Postoperative plan. VAS: visual analog scale.

Time	Immediate postoperative	1 hour	2 hours	6 hours	12 hours	24 hours
VAS						
Rescue analgesics						

Blood Glucose Levels

**Table 14:** Blood glucose levels.

Time point	Value
Preoperative	
Intraoperative	
Immediate postoperative	
After 6 hours	

Postoperative Nausea/Vomiting

YES/NO

If YES, grading of nausea/vomiting:

**Table 15:** Postoperative nausea/vomiting.

Severity	Description
Mild (only nausea)	
Moderate (nausea & vomiting)	
Severe (multiple episodes of vomiting)	

If severe, number of episodes:

Time from end of surgery:

Treatment:
